# Progress on the roles of MEF2C in neuropsychiatric diseases

**DOI:** 10.1186/s13041-021-00892-6

**Published:** 2022-01-06

**Authors:** Zhikun Zhang, Yongxiang Zhao

**Affiliations:** 1 National Center for International Research of Bio-Targeting Theranostics, Guangxi Key Laboratory of Bio-Targeting Theranostics, Collaborative Innovation Center for Targeting Tumor Diagnosis and Therapy, Guangxi Medical University, Nanning, 530021 Guangxi China; 2 Department of Mental Health, The Second Affiliated Hospital of Guangxi Medical University, Nanning, 530007 Guangxi China

**Keywords:** MEF2C, Transcription factor, Synapse, Neurodevelopment, Neuropsychiatric disease

## Abstract

Myocyte Enhancer Factor 2 C (MEF2C), one of the transcription factors of the MADS-BOX family, is involved in embryonic brain development, neuronal formation and differentiation, as well as in the growth and pruning of axons and dendrites. MEF2C is also involved in the development of various neuropsychiatric disorders, such as autism spectrum disorders (ASD), epilepsy, schizophrenia and Alzheimer’s disease (AD). Here, we review the relationship between MEF2C and neuropsychiatric disorders, and provide further insights into the mechanism of these diseases.

## Introduction

MEF2C is an important member of the myocyte enhancer factor 2 (MEF2). MEF2 is a subfamily of the MADS-BOX (MCM-1-agamous-deficiens-serum response factor) family of transcriptional regulatory factors, which play essential roles in embryogenesis and epigenetic modifications that control gene expressions during development and throughout adulthood [23]. The MEF2 family consist of four members MEF2A, MEF2B, MEF2C and MEF2D. MEF2C is the earliest expressed MEF2 isomer in the telencephalon of mouse embryos, and is the most expressed in the cerebral cortex of postnatal and adult mouse brains. Therefore, it is critical for proper nervous system development and functional maintenance [4].

The human MEF2C gene is located in the chromosome 5q14.3 region and its protein consists of five core domains; MADS domain, MEF2 domain, transcriptional activation domains 1 (TAD1) and 2 (TAD2), as well as the nuclear localization signal (NLS). The MADS and MEF2 domains mediate MEF2C dimerization, DNA binding as well as recruitment of cofactors. TAD is involved in the recruitment of cofactors, including co-activators such as histone acetyltransferase p300 and cAMP-response element-binding protein-binding protein (CBP), or co-repressors such as class II histone deacetylases (HDACs), that regulates transcription [4]. Besides, through alternative splicing at the mRNA level [85], and post-translational modifications such as phosphorylation or dephosphorylation [11], acetylation [3], sumoylation [63] and S-nitrosylation [52], MEF2C appears various expression pattern and transactivation functions. In addition, MEF2C mediates physiological processes such as cardiac morphogenesis, angiogenesis, muscle cell differentiation, bone development, and neural or lymphatic system development [44].

**Fig. 1:**
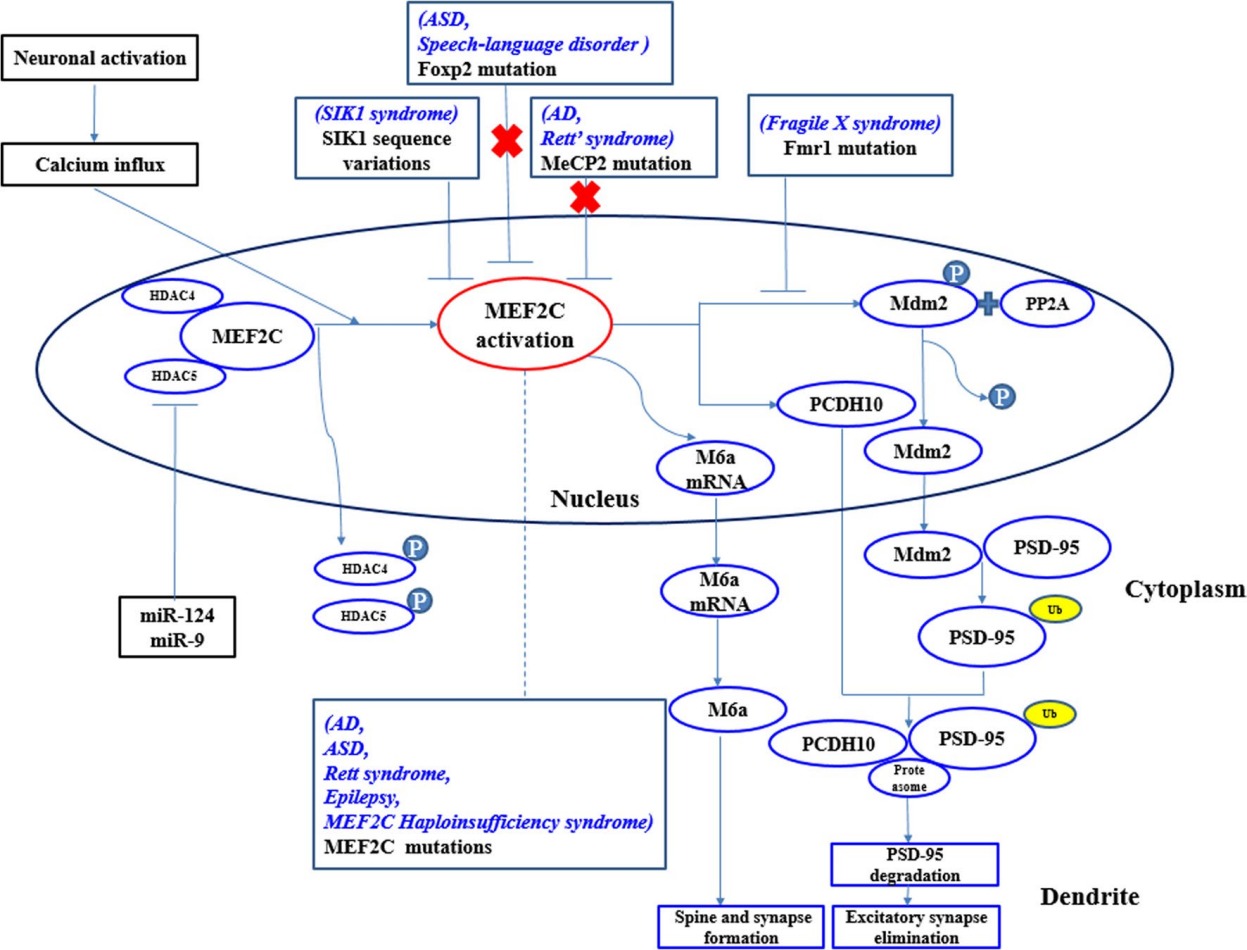
The functional regulation model of MEF2C in neuronal cells, where MEF2C is particularly important for the regulation of dendritic spines. The blue italics are MEF2C-associated psychoneurotic disorders

In the absence of external stimulation, the MEF2 protein in the central nervous system binds and inactivates target genes. Various stimuli, such as depolarization, neurotrophin or synaptic stimulation (e.g., glutamate synaptic Reelin) activates neurons, thereby triggering calcium signaling responses, including calmodulin-dependent protein kinase (CaMK), leading to phosphorylation of class IIa HDACs. These phosphorylation events cause to a nuclear output of HDACs, and their subsequent removal from the MEF2 target gene, allowing the recruitment of co-activators [46]. MiR-124 and miR-9 have been shown to co-inhibit HDACs 5 expressions, which activate the neuron membrane glycoprotein, GPM6A, a MEF2C target gene. These activities promote the development of axons in primary neurons (Fig. 1) [25].

In addition, protein phosphatase 2B or calcineurin can directly dephosphorylate MEF2, thereby affecting its affinity towards target DNA sequences, and promoting its transcription activities [43]. MEF2C is involved in regulation of neuronal migration, activity-dependent cell survival, neuronal differentiation, axon guidance and pruning, dendritic formation and remodeling, as well as in synaptic development and neuronal excitability [37, 39, 84]. In the last decade, human genome-wide association study (GWAS) and genome sequencing of large patient samples revealed that MEF2C is a candidate risk gene for various neuropsychiatric disorders, such as AD [67], ASD [83], schizophrenia [47], bipolar disorder [50], major depression [28], attention deficit and hyperactivity disorder [62], epilepsy [80] or Parkinson’s disease [59]. We review recent findings on MEF2C as well as its role in some neuropsychiatric diseases and provide theoretical and detailed insights that will inform future studies.

### Alzheimer’s disease

AD is a neurodegenerative disease that is associated with progressive cognitive and memory deterioration. Inflammation is involved in AD pathogenesis [19]. As the first and main immune defense component of central nervous system (CNS), microglia act as macrophages, clearing metabolites and necrotic cells in the brain through phagocytosis. However, continuous activation of microglia leads to the secretion of various neurotoxic substances, which play an important role in AD development [54]. For instance, when interferon-β (IFN-β), a pro-inflammatory cytokine related to brain ageing, was injected into the cerebrospinal fluid of young wild type (WT) or MEF2C-knock out (MEF2C-KO) mice, suppressed social interaction outcomes were observed in the MEF2C-KO mice, compared to WT mice. Following immune activations, the expressions of pro-inflammatory chemokines (CCL2 and CCL5) and cytokines (IL-1b and TNF) in MEF2C-KO mice were significantly higher than in WT mice. Thus MEF2C enhances the resilience of microglia to immune challenges and promotes homeostasis in pre-inflammatory states. Type I interferon (IFN-I) accumulation down-regulates the MEF2C in microglia, leading to excess microglia responses under pro-inflammatory environments of AD or aging brains, and aggravates cognitive impairment and disease pathology [20]. As reported by Xue et al., the suppressed MEF2C nuclear translocation as an early and prominent microglial phenotype in a mouse model of brain amyloidosis (5 × FAD mice) implies an association between MEF2C deregulation and microglial phenotypes in AD-relative settings. Although they did not report the downregulation of MEF2C mRNA copies in the microglia of 5 × FAD mice, upregulated MEF2C in microglia after anti-IFNAR antibody administration implies a potential strategy for improving microglial elasticity by upregulation of MEF2C [77]. A significant decrease in MEF2C mRNA levels in leukocytes of AD patients has been positively correlated with total scores of Mini-Mental State Examination (MMSE). Therefore, expression levels of MEF2C mRNA in leukocytes may not only be a biomarker for AD diagnosis, but be a potential biomarker for early detection of cognitive decline, such as mild cognitive impairment (MCI) [60].

In addition, MEF2C plays an important role in amyloid-protein precursor protein (APP) -mediated anti-apoptotic neuroprotection [12]. MEF2C is a potential regulator of APP proteolysis, during which amyloid beta (Aβ), a central factor in AD initiation, is produced [13]. These findings elucidate on pathways involved in oligomeric Aβ-potentiated microglial activation.

Methyl CpG binding protein 2 (MeCP2; an important transcriptional inhibitor) levels have been shown to be increased in the cerebral cortex and hippocampus of AD patients. MeCP2-mediated MEF2C inhibition may be associated with cognitive decline in AD [33]. Interestingly, other studies have reported a significant correlation between cognitive functions and seasons among the elderly. There is a 4-year difference in cognitive functions between summer and autumn, compared to winter and spring, while the probability of meeting the criteria for MCI or dementia in winter and spring is nearly 30% higher, relative to that in the summer and autumn. MEF2C was found to be involved in this regulation of seasonal plasticity [40].

The high expressions of MEF2C in brain regions related to learning and memory, such as dentate gyrus, frontal cortex, entorhinal cortex or amygdala, strongly prompted the important role of MEF2C in cognition of AD [81]. Large meta-analysis studies identified that the variation of rs190982, a common single nucleotide polymorphism (SNP), in MEF2C is a protective factor against AD in a Caucasian population (OR = 0.93, 95% CI = 0.90 ~ 0.95) [66]. Besides, an association between rs190982 polymorphism and AD (OR = 0.885, 95% CI = 0.811 ~ 0.966) has been reported [10]. Based on analyses of pathological data on AD, among 22 genetic variations of AD, the rs190982 variation in MEF2C gene, was the only one that was found to exert significant effects on cognitive functions [42]. However, other studies have reported the negative association [8, 66]. A genome-wide association studies (GWAS) meta-analysis involving a Taiwanese population of China did not reveal positive association between MEF2Crs190982 polymorphism and AD, however, there were interactions between MEF2Crs9293506 and cognitive aging. The risk for cognitive aging in carriers with TC genotypes of MEF2C rs9293506 was 2.79 times higher than that of those with the CC genotype [41]. These variations may be attributed to genetic heterogeneities among different ethnic groups, such as differences in minor alleles, different frequencies of secondary alleles or heterogeneity of potential genetic structures.

In a 2013 meta-analysis, Lambert et al. found a genome-wide significant association between MEF2C and AD [35], however, the significant association was not replicated in a larger sample study performed in 2019 [34]. Moreover, Jansen et al. were unable to establish an association between MEF2C and AD in a large sample study from the UK Biobank (UKB) [29].

Although GWAS have been a valuable platform for identifying candidates for disease-related genetic variants, the confirmed risk loci for AD only explains a small portion of AD heritability (Table 1). To address some of the limitations encountered by univariate SNP-based analyses, advanced methods were developed to examine SNPs in aggregate. The aggregated SNP approach may reduce the total number of tests performed and increase power by exploiting linkage disequilibrium (LD) across multiple SNPS [76]. It hypothesized that variations in entire gene regions, rather than isolated single SNPS, play a role in cognitive decline [48]. Other strategies for improving detection effects should be evaluated in large sample sizes composed of different ethnic populations.

**Table 1:** MEF2C single nucleotide polymorphisms with Alzheimer's disease in different population

Sample	MEF2C Genotype	Phenotype	References
Caucasian population	SNPrs190982	Protective factor	[35]
Spanish population	SNPrs190982	Protective factor	[58]
Han Chinese	SNPrs190982	Effect factor on cognition	[42]
Han Chinese	SNPrs190982	No association	[66]
Alzheimer’s Research UK Consortium DNA Bank	SNPrs190982	No association	[8]
Taiwanese	SNPrs190982	No association	[41]
Taiwanese	SNPrs9293506	TC genotype had a 2.79-fold increased risk for cognitive aging compared to CC genotype	[41]
Japanese	MEF2C mRNA in leukocytes	Positively correlated with MMSE	[60]

*MMSE* Mini-mental State Examination, *SNP* single nucleotide polymorphisms

### Epilepsy

Due to involvement of MEF2C in many processes during neural developmental stages, functional disruption of MEF2C results in various neurological symptoms, among which epilepsy is a common symptom. MEF2C conditional knockout mice revealed that MEF2C is involved in the migration of GABA and glutamate pyramidal neurons, as well as in the maintenance of synaptic stability and function [71]. Besides, MEF2C regulates inhibitory and excitatory states of neurons to maintain the balance in neural networks [26]. Disruption of this regulation may lead to abnormal synaptic activities, causing epileptic events.

MEF2C-related epilepsy has been reported [9, 53, 82] (Table 2). Its prevalence ranges between 54% and 82% [9, 53]. The associated genetic defects include MEF2C pathogenic variants, or microdeletions encompassing the MEF2C gene. MEF2C-related epilepsy usually occurs during infancy or early childhood. In a review involving 19 patients with MEF2C-related epilepsy, seizures were established to have occurred in the first 12 months of life in 12 patients (63%). The mean age of seizure onset was 13.5 months (median 12), ranging from 3 to 36 months [9]. Febrile epilepsy, tonic–clonic epilepsy and myoclonic epilepsy are the most common epilepsy types. Reported EEG abnormalities include epileptic activity, background activity disorder, and multifocal as well as generalized epileptiform discharge [9]. Although the frequency of early-onset epilepsy is high, some children with late onset, mild seizures or no epilepsy at all exhibit serious neurological defects, such as low muscle tone or hyperactivity. The severity of epilepsy is not always consistent with other neurological defects [49]. Certainly, patients with complete deletion of MEF2C have an increased risk for developing epilepsy, relative to those with partial deletion [57].

**Table 2:** Genomic data and key features of epilepsy for 42 patients with MEF2C-relative epilepsy

	Genetic defect	Epilepsy phenotype	References
1	De novo, likely pathogenic heterozygous variant, MEF2C: c.236 G > C (p.Arg79Pro)	Focal impaired awareness motor seizures	[9]
2	De novo, missense variant, MEF2C: c.48C > G (p.Asn16Lys)	Focal seizures	[75]
3	Pathogenic heterozygous variant, MEF2C: c.565C > T (p.Arg189)	Not reported	[75]
4	Heterozygous variant, MEF2C: c.334 G > T (p.Glu112)	Focal seizures	[75]
5	De novo, heterozygous variant, MEF2C: c.403-1 G > T	Febrile seizures, followed by afebrile seizures	[75]
6	De novo, pathogenic heterozygous variant, MEF2C: c.766C > T (p.Arg256)	Febrile seizures	[75]
7	5q14.3q15 del, GC Chr5: 88 098 253-88 592 348	Febrile seizures	[73]
8	5q14.3q15 del, GC Chr5: 88 034 622-88 164 453	Febrile seizures, followed by generalized seizures	[73]
9	5q14.3q15 del, GC Chr5: 88 193 289-88 450 318	Febrile seizures, followed by generalized and absence seizures	[73]
10	De novo, pathogenic heterozygous variant, MEF2C: c.220 G > T (p.Glu74Ter, premature stop codon)	Febrile seizures and afebrile seizures	[73]
11	MEF2C deletion, exons1–2 (MLPA)	Not reported	[73]
12	De novo, pathogenic missense heterozygous variant, MEF2C: c.9A > T (p.R3S)	Atypical absence, atonic, myoclonic and refractory seizures	[57]
13	5q14.3 del (0.01 Mb), GC Chr5: 88 110 707–88 278 367	Not reported	[68]
14	De novo, missense heterozygous variant, MEF2C: c.258 G > A (p.E86E)	Not reported	[65]
15	Pathogenic frameshift variant, MEF2C: c.833delT (p.Leu278Terfs)	Myoclonic and atonic seizures	[53]
16	5q14.3 del (0.05 Mb), GC Chr5: 880 519 70-881 045 35	Not reported	[53]
17	Pathogenic frameshift heterozygous variant, MEF2C c.457delA (p.Asn153ThrfsX33)	Myoclonic and febrile seizures	[7]
18	5q14.3 del (3.6 Mb), GC Chr5: 85,855,118–89,474,751	ISS	[53]
19	5q14.3 del (5.11 Mb), GC Chr5: 85,684,257–90,798,560	Myoclonic epilepsy	[53]
20	5q14.3 del (1.0 Mb), GC Chr5: 88,018,766–89,063,989	Not reported	[53]
21	5q14.3 del (1.38 Mb), GC Chr5: 87,905,325–89,289,023	Myoclonic epilepsy, followed by ISS	[53]
22	5q14.3 del (0.32 Mb), GC Chr5: 87,905,325–88,220,403	Myoclonic and generalized epilepsy	[53]
23	Frameshift Mutation in MEF2C, c833delT	Myoclonic and atonic epilepsy	[53]
24	5q14.3 del (1.95 Mb), GC Chr5: 87,566,009–89,505,509	Myoclonic epilepsy and ISS	[53]
25	5q14.3 del (6.0 Mb), GC Chr5: 87,719,139–93,736,389	ISS	[53]
26	5q14.3 del (11.6 Mb), GC Chr5: 81,657,245–93,240,731	Febrile seizures	[53]
27	5q14.3 del (5.4 Mb), GC Chr5: 88,185,348–93,546,896	Myoclonic epilepsy	[53]
28	5q14.3 del (0.41 Mb), GC Chr5: 88,177,038–88,592,311	Febrile seizures	[53]
29	5q14.3 del (5.2 Mb), GC Chr5: 84,520,000–89,800,000	Myoclonic epilepsy	[53]
30	De novo, pathogenic missense heterozygous variant, MEF2C: c.113T > A (p.Leu38Gln)	Not reported	[86]
31	De novo, heterozygous 1-bp duplication of the MEF2C gene: 99dupT (p.E34X)	Complex partial seizure	[86]
32	Pathogenic variant, MEF2C: c.226_236del11 (p.H76fsX15)	Not reported	[86]
33	De novo, heterozygous missense variant, MEF2C:c.80 G > C (p.Gly27Ala)	Not reported	[86]
34	De novo, heterozygous nonsense variant, 683C-G transversion in exon 7 of the MEF2C gene	Not reported	[36]
35	5q14 del (0.02 Mb), GC Chr5: 87 770 283-88 051 970	Febrile seizures	[36]
36	5q14.3 del (3.24 Mb), arr5q14.3q15 (890 687 77-923 160 85) × 1, hg19	ISS	[10]
37	5q14.3 del (5.69 Mb), arr cgh 5q14.3q15 (rs10514301 − rs9314105) × 1 dn	ISS, occasional seizures	[22]
38	5q14.3 del (3.6 Mb), arr cgh 5q14.3 (RP11-291O24-RP11-62E10) × 1 dn	Febrile seizure	[22]
39	5q14.3-q15 del (3.574 Mb), arr cgh 5q14.3q15 (rs10223241 − rs17664587) × 1 dn	Atypical absences, followed by complex partial seizures	[22]
40	5q14.3-q21.3 del (17 Mb), GC Chr5:88 945 075–105 929 555	Febrile seizures, followed by generalized tonic–clonic seizures	[14]
41	5q14.3-q15 del (8.4 Mb), GC Chr5: 87 086 298–95 538 699	ISS, epileptic spasms	[14]
42	5q14.3-q15 del (6.3 Mb), GC Chr5: 88 659 488–94 986 600	Episodes of unresponsiveness, followed by myoclonic seizures	[14]

*Del* deletion, *GC* genomic coordinates, *Mb* megabase, *ISS* infantile spasms

Etiopathogenic factors in other conditions are also associated with MEF2C in epilepsy. Among 73 patients with infantile spasm syndrome (ISS), an age-related epileptic syndrome, one patient with 3.24 Mb deletion in 5q14.3 located in 1 Mb upstream of the MEF2C gene was found [arr5q14.3q15 (890, 687, 77 ~ 923, 160, 85) x1, hg19] [10]. Bienvenu et al. identified a case of de novo MEF2C mutation in 50 patients with unexplained epileptic encephalopathy [7]. Moreover, Yu et al. and Zhou et al. reported cases of infantile spasm caused by a 5q14.3 microdeletion syndrome in China [79, 82]. Another study documented a 9.68% rare functional variation of MEF2C in ASD patients with epilepsy, but not in ASD patients without epilepsy, indicating that MEF2C functional variation significantly increased the risk of epilepsy in ASD [83].

In recent years, molecular studies have revealed further pathological mechanisms involved in epilepsy. For instance, truncated Salt-induced kinase 1 (SIK1) sequence variants p. (Glu347*) and p. (Gln633*) reduced the expressions of MEF2C. The SIK1 syndrome is a developmental epilepsy disorder that is caused by a heterozygous mutation in the SIK1 gene (OMIM no.616341). Decreased expressions of MEF2C proteins in neurons is correlated with abnormal expressions of target genes (ARC, NRG1 or NR4A1), which disrupts the balance in neuronal excitability, thereby reducing the epileptic threshold. This process is independent of HDAC5 phosphorylation, indicating that SIK1 may directly interact with MEF2C [55]. In addition, in rat models, the expressions of miR-203 in astrocytes were established to be up-regulated, which led to down-regulation of MEF2C, promotion of NF- κB, phosphorylation of I κ B/IKK and secretion of inflammatory effectors (IL-6 and TNF-α). Besides, LncRNAUCA1 (long-chain non-coding RNA urothelial carcinoma associated 1) inhibits inflammatory responses to epilepsy by modulating miR203-mediated regulation of the MEF2C/NF-κB signaling pathway, therefore, it may be a potential therapeutic target for epilepsy [80].

### Nervous system tumor

The activities of MEF-2 are regulated by various factors, including alternative splicing, post-translational modification of C-terminal and dimerization of N-terminal with other transcription factors [44]. Not only are mutations in these regions associated with developmental abnormalities, they are also associated with tumors, leukemia and transcriptional abnormalities. MEF2C diversifies hematological tumors, pancreatic cancer or liver cancer, leading to exhibition of various tumor characteristics [17]. MEF2C plays an important role in tumor pathogenesis and development, however, a limited number of studies have evaluated its functions in nervous system tumors [21, 78].

A study on brain metastases of breast cancer (BCBM) demonstrated that MEF2C, as a target gene of miR-802-5p and miR-194-5p, is increased in metastatic tumor cells. Immunoreactivity analyses showed that MEF2C expression increased by 24% between the 3rd and 10th day of brain metastasis (p < 0.001), and by 20% between the 7th and 10th day (p < 0.001). Therefore, as a transcription factor, MEF2C promotes the development of metastatic tumors. It was also found that peritumoral astrocytes began to express MEF2C after exudation of tumor cells from tumor tissues, while non-peritumoral astrocytes did not show these expressions, suggesting that MEF2C is involved in the crosstalk between astrocytes and tumor cells during the development of BCBM  [61].

In the cytoplasm, MEF2C was shown to impair β-catenin translocation into the nucleus, thereby inhibiting Wnt/β-catenin signaling during the early stages of metastases development. However, in advanced stages, MEF2C and Wnt/β-catenin translocated to the nucleus, which was accompanied by an increase in Ki-67 positive cells. Continuous expression of MEF2C and its translocation to the nucleus is associated with disease severity, and MEF2C may serve as a biomarker for BCBM development and prognosis as well as its potential therapeutic target [24].

The dual role of MEF2C in tumors has also been reported in liver cancer. In the nucleus of hepatocellular carcinoma, MEF2C promoted the invasion and angiogenesis of hepatocellular carcinoma cells, while cytoplasmic MEF2C isolated β-catenin in the cytoplasm and reduced the ability of β-catenin to promote cell proliferation. Subcellular distribution of MEF2C may determine the overall role of MEF2C [5]. On the other hand, after being assembled into multi-protein complexes as transcription factors, MEF2C can be transformed into transcription activators or inhibitors under the control of tumor microenvironments to produce the opposite effects [44]. Therefore, MEF2C may act as a “double-edged sword” (either as a proto-oncogene or a tumor suppressor) in tumor pathogenesis.

### Autism spectrum disorders

Autism spectrum disorders (ASD) are a group of severe neurodevelopmental disorders that are characterized by impaired social interactions and communication skills, and/or narrow interests and repetitive stereotyped behaviors. Although there are many etiological models for ASD, such as gene mutations, abnormal synaptic development or signaling pathways, its pathogenesis has not been conclusively determined. One of the mechanisms involved in ASD pathogenesis is the imbalance in excitatory/inhibitory synaptic ratio [15]. Synaptic formation dominates the early stages of brain development, resulting in the generation of more synapses than are needed for brain functions. Consequently, the brain prunes the extra synapses [27]. MEF2C plays an important role in activity-dependent synaptic elimination. When depolarization and calcium influx occur in neurons after stimulation, MEF2C is activated, then it induces the transcription of protocadherin-10 (Pcdh10). Pcdh10 mediates the degradation of synaptic scaffold protein 95 (PSD-95) by binding ubiquitin PSD-95 to the proteasome, leading to elimination of excitatory synapses [64, 69].

Dysfunctions of the MEF2C gene may prevent the brain from eliminating unwanted excitatory synapses, leading to ASD-like syndromes (Table 3). A previous study reported a significant increase in the number of excitatory synapses and spinous processes in MEF2C-KO mice, as well as enhancement of basal and evoked synaptic transmission. This cascade of events led to development of hippocampal dependent learning and memory impairments, as well as ASD-like social behavior defects [6]. Conditional MEF2C KO in neural stem/progenitor cells expressing Nestin can affect neuronal differentiation, resulting in abnormal density and cell body sizes of cortical plate neurons, without affecting the proliferation as well as survival of neural stem cells. The conditioned MEF2C-KO mice that survived to adulthood showed more immature electrophysiological network characteristics and serious behavioral defects, indicating that MEF2C plays a key role in early programming of neuronal differentiation and distribution of neocortical layers in ASD [5]. Unlike the previous thought that MEF2C was only expressed in the cerebral cortex and hippocampus [2], recent studies have found that MEF2C was  specifically expressed in the Purkinje cell layer of the cerebrum [32]. It selectively regulates the development of dendrites of Purkinje cells and prunes the synapses of climbing fibers. Similar with ASD findings, deletion or downregulation of MEF2C resulted in increased dendritic branches and spines in Purkinje cells, and changes in excitatory as well as inhibitory synaptic protein localization [37, 39]. Essentially, the increased spines are immature spines that are most likely to be pruned later, resulting in a decrease in the number of neurons and a decline in overall functions [45].

**Table 3:** Neural phenotype and behavior phenotype in manipulation of MEF2C

Sample	Neural phenotype	Behavior phenotype	References
Calcium/calmodulin-dependent protein kinase II (CaMKII)-Cre93 line conditioned MEF2C-KO mice after birth	Increased the number of spines in the hippocampus of mice	Not related with the presentation of learning and memory, LTP or social behavior	[16]
Late embryonic deletion of MEF2C in the forebrain Transgenic expression of a superactive form of MEF2C in neurons of mice (NSE-MEF2C-VP16 transgenic mice)	Increased the number of excitatory synapses and spinous processes Enhanced basal and evoked synaptic transmission Reduced structural and functional glutamatergic synapse density in hippocampal pyramidal neurons	Hippocampus-dependent learning and memory impairment	[6]
Conventional exon 2-deleted allele of MEF2 deletion or downregulation of MEF2C	Increased dendritic branches and spines in Purkinje cells, and changes in excitatory and inhibitory synaptic protein localization		[37, 39]
Conditional MEF2C gene KO in neural stem/progenitor cell	Abnormal density and cell body size of cortical plate neurons	More immature electrophysiological network characteristics and serious behavioral defects	[5]
Knockdown of MEF2C Overexpression of MEF2C	Reduced the number of dendritic spines on apical dendrites of cultured neural progenitor cells Increased spine density	Hyper-sensitive passive avoidance behavior	[31]
Embryonic MEF2C deletion from most forebrain excitatory neurons in mice (EmxCre × MEF2C flox/flox)	A ~ twofold increase in dendritic GABAergic synapse density on excitatory cortical pyramidal neurons	Deficits in fear learning and memory, multiple social behaviors, socially-motivated ultrasonic vocalizations, and reward-related behaviors	[26]
HSV-Cre-GFP virus injection in MEF2C flox/flox pups at P2 to down-regulate MEF2C expression HSV-Cre-GFP virus injection in MEF2C flox/flox mice at P14-15 to down-regulate MEF2C expression In utero electroporation of pcBIG-Mef2C-VP16 plasmids at E12.5 in wild-type embryos to overexpress MEF 2C	Increased in spine counts in SPNs at P8 Normal number of dendritic spines in SPNs at P19-20 Decreased number of spines in SPNs at P14	Defective neonatal isolation-induced USVs, a form of vocal communication in neonatal rodents	[18]
Postnatal MEF2C deletion AAV-Cre-GFP infection in dissociated neocortical cultures	Decreased excitatory synapse number from L4 / L2/3 pyramidal neurons A reduced spine density on basal of normal dendritic branching in neurons		[56]
Conventional exon 2-deleted allele of MEF2C	Reduced number of neurons and total dendritic lengths Dendritic interactions impairment Increased E/I ratio in the hippocampus	Intellectual disability, speech deficit, autism-like symptoms, seizures or motor abnormalities	[70]
Upregulation of MEF2C in the adult prefrontal cortex (PFC) by AAV-MEF2C virus injections	Decrease in mushroom spines proportion in layer III of the PFC with no difference in total spine density	Improved cognition	[47]

*SPNs* striatal projecting neurons

Studies on deletions of MEF2C in frontal brain regions of conditioned MEF2C-KO mice after birth, using the calcium/calmodulin-dependent protein kinase II (CaMKII)-Cre93 line, showed that although the number of spines in the hippocampus of mice increased significantly, these increases were not related with presentation of learning and memory, long-term potentiation (LTP) or social behaviors [16]. In spite of continuous roles of MEF2C in negative regulation of synaptogenesis, the functions of MEF2C, which involves the regulation of synaptic plasticity, learning and memory or behavioral expressions in ASD may depend on expressions of MEF2C during embryonic development, rather than just regulation of the number of synapses [1].

There is a significant overlap between the genes regulated by MEF2C and dozens of candidate ASD risk genes [26]. For instance, ASD-related symptoms have been reported in some patients with MEF2C haploid insufficiency syndrome (MCHS) [72]. MCHS, a neurodevelopmental disorder, is caused by microdeletion, missense or nonsense mutations in the MEF2C gene or copy number variations (CNVs). Chromosomal microarray data revealed that MEF2C mutations are scattered throughout the MEF2C protein, without thermal mutation regions. The patients showed varying degrees of intellectual disabilities, speech deficits, autism-like symptoms, seizures or motor abnormalities [36]. In general, phenotypic characteristics of MCHS may present dysplasia of multiple nerve cell populations at the transcriptional level, with ASD syndromes being the most pronounced [57].

Rett syndrome, one of the serious neurodevelopmental diseases, which occurs mostly in women. It is characterized by progressive decline in motor skills and intelligence. The MeCP2 mutation allele in region q28 on X chromosome has been shown to trigger the Rett syndrome [38]. Jiaping W et al. found 3 MEF2C heterozygous mutations in 44 patients with the Rett syndrome, without MeCP2 gene mutations, suggesting the MEF2C gene mutation is one of the risk factors for Rett syndrome [74]. Based on definitive pathogenesis of MeCP2 gene mutations, DSM-5 removed Rett syndrome from the diagnosis of ASD in 2013. However, this change has been questioned by many scholars. Since ASD is diagnosed via language as well as behavioral symptoms and is also caused by genetic variations, it may not remove a subtype of ASD when one pathogenic gene is found. According to current studies, some of the patients diagnosed with Rett syndromes do not have the MeCP2 gene mutation, but share common pathogenic genes with ASD. Therefore, more evidence is needed to distinguish Rett syndrome from ASD.

### Schizophrenia

Schizophrenia is a severe, disabling mental disorder. Cognitive impairments in schizophrenia related to the prefrontal lobe are highly associated with disability. In a study involving 150,000 participants, the Psychiatric Genome Association identified 108 different genome loci associated with schizophrenia [51]. There are MEF2C target sites in the heritable risk factors for schizophrenia, which associate MEF2C with this disease.

An enrichment of MEF2C motifs in the SNP pool, with the top score being related to schizophrenia was reported [47]. Sequencing data of chromatin-associated histone methylation in the prefrontal neuronal chromatin of 17 schizophrenic patients showed that MEF2C-binding motifs were significantly overexpressed in about 1000 nucleosome sequences, affected by histone H3K4 hypermethylation. The hypermethylated sequence of trimethyl-histone H3-lysine 4 (H3K4me3) exhibited a strong neuronal footprint, with 6/12 of Gene Ontological (GO) categories being associated with synapses and neurons, and 8/18 of “drug” and “phenotypic” categories being matched with decreased cognition and abnormal behaviors. Down-regulation of MEF2C in cell culture models showed hypermethylation of H3K4 in affected nucleosomes, similar to the changes observed in prefrontal lobes of schizophrenia. Increasing expressions of MEF2C in the prefrontal lobe of cognitive impaired mice models with schizophrenia by using adeno-associated virus vectors significantly improved cognitive abilities. Following treatment with the NMDA receptor antagonist, MK-801, the cognitive performance of mice with up-regulated MEF2C levels were better compared to those of normal mice. Therefore, MEF2C transcription factors are promising targets for treatment of schizophrenia-associated cognitive impairment [30].

### Summary

MEF2C plays a significant role in early brain development of humans or animals, and in normal development, distribution and electrical activity of neocortical neurons. Besides, it has a profound effect on neuropsychiatric phenotypes. Because of the important effects of MEF2C on synapses, MEF2C gene mutations or dysfunctions will lead to a series of syndromes, including intellectual deficiency, epilepsy and autism-like symptoms. The association between MEF2C and cognitive impairment coincides with the role of MEF2C in AD and ASD. In view of similar symptoms caused by MEF2C defects in various neuropsychiatric disorders, we recommend that it be described as MEF2C-related syndrome, which would be contribute to identify characteristic gene-related symptoms from these complex neuropsychiatric disorders. Further research on MEF2C will elucidate on the pathogenesis of neurological and mental disorders, as well as provide insights for improvement of symptoms associated with MEF2C gene deficiencies in neuropsychiatric disorders.

Tu et al. [70] reported that MEF2C heterozygous KO mice exhibited intellectual disabilities, autism-like symptoms, seizures, as well as motor abnormalities. After continuous injection of NitroSynapsin (a new antagonist of NMDA-type glutamate receptors) for 3 months, mice autism-like symptoms were significantly improved. These protective effects were associated with prevention of the loss of neurons and correcting the ratio of excitatory to inhibitory (E/I) neurotransmission imbalance [70]. Besides, in mice, neuronal MEF2C overexpression in adult prefrontal cortex improves working memory and object recognition memory, in conjunction with spinal remodeling in prefrontal projection neurons [47]. Therefore, these results have important implications in treatment of ASD, schizophrenia and other MEF2C-related neuropsychiatric disorders, at least improve the symptoms associated with MEF2C.

## Data Availability

Data sharing not applicable to this article as no datasets were generated or analysed during the current study.
